# Genetic influences and causal pathways shared between cannabis use disorder and other substance use traits

**DOI:** 10.1038/s41380-024-02548-y

**Published:** 2024-04-05

**Authors:** Marco Galimberti, Daniel F. Levey, Joseph D. Deak, Hang Zhou, Murray B. Stein, Joel Gelernter

**Affiliations:** 1 Department of Psychiatry, Yale University School of Medicine, New Haven, CT, USA.; 2 Veterans Affairs Connecticut Healthcare System, West Haven, CT, USA.; 3 Department of Psychiatry and School of Public Health, University of California San Diego, La Jolla, CA, USA.; 4 VA San Diego Healthcare System, San Diego, CA, USA.; 5 Departments of Genetics and Neuroscience, Yale University School of Medicine, New Haven, CT, USA.

## Abstract

Cannabis use disorder (CanUD) has increased with the legalization of the use of cannabis. Around 20% of individuals using cannabis develop CanUD, and the number of users has grown with increasing ease of access. CanUD and other substance use disorders (SUDs) are associated phenotypically and genetically. We leveraged new CanUD genomics data to undertake genetically-informed analyses with unprecedented power, to investigate the genetic architecture and causal relationships between CanUD and lifetime cannabis use with risk for developing SUDs and substance use traits. Analyses included calculating local and global genetic correlations, genomic structural equation modeling (genomicSEM), and Mendelian Randomization (MR). Results from the genetic correlation and genomicSEM analyses demonstrated that CanUD and cannabis use differ in their relationships with SUDs and substance use traits. We found significant causal effects of CanUD influencing all the analyzed traits: opioid use disorder (OUD) (Inverse variant weighted, IVW *β* = 0.925 ± 0.082), problematic alcohol use (PAU) (IVW *β* = 0.443 ± 0.030), drinks per week (DPW) (IVW *β* = 0.182 ± 0.025), Fagerström Test for Nicotine Dependence (FTND) (IVW *β* = 0.183 ± 0.052), cigarettes per day (IVW *β* = 0.150 ± 0.045), current versus former smokers (IVW *β* = 0.178 ± 0.052), and smoking initiation (IVW *β* = 0.405 ± 0.042). We also found evidence of bidirectionality showing that OUD, PAU, smoking initiation, smoking cessation, and DPW all increase risk of developing CanUD. For cannabis use, bidirectional relationships were inferred with PAU, smoking initiation, and DPW; cannabis use was also associated with a higher risk of developing OUD (IVW *β* = 0.785 ± 0.266). GenomicSEM confirmed that CanUD and cannabis use load onto different genetic factors. We conclude that CanUD and cannabis use can increase the risk of developing other SUDs. This has substantial public health implications; the move towards legalization of cannabis use may be expected to increase other kinds of problematic substance use. These harmful outcomes are in addition to the medical harms associated directly with CanUD.

## INTRODUCTION

Cannabis use disorder (CanUD) is increasing in importance worldwide. A national survey of 36,309 participants ≥18 years old showed 12-month and lifetime CanUD prevalence of around 2.5% and 6.3% in the United States, respectively [1]. In general, substance use disorders (SUDs) cause downstream social problems, such as driving under the influence and road accidents, partner and sexual violence, child abuse and neglect [2], and occupational impairments. SUDs also have direct negative impacts on health, raising risk for cardiovascular diseases [3, 4], cancer [5], stroke [6], and premature death from overdose [7] among other harms, and may also exacerbate and result in the development of other mental disorders [8]. The relationship of CanUD to other SUDs is an important public health issue, and could amplify the harms attributable to CanUD; CanUD specifically has already been found associated to lung cancer [9], reduced cognitive ability [10], and increase of other mental disorders [9].

Risk of developing CanUD increases with daily use of cannabis [11] and by use of more potent cannabis products [12]. A meta-analysis demonstrated a risk of 20% for developing CanUD among cannabis users, increasing to around 30% for individuals who use cannabis at least weekly [13]. The trend towards legalization of medical and recreational cannabis use has led to increased use [14, 15]. Co-twin analyses have shown that CanUD risk lightly increases with cannabis use frequency [16]. Thus, cannabis legalization increases cannabis use frequency, a prerequisite to CanUD. Genetic studies have found a moderate genetic correlation (r_g_) between CanUD and cannabis use (r_g_ = 0.50 ± 0.05, *p* = 1.5 × 10^−21^) [17]. Furthermore, CanUD and cannabis use have significant differences in their genetic correlations with numerous other traits [9]. In particular, alcohol use disorder, cigarettes per day, Fagerstrom test for nicotine dependence (FTND), and smoking initiation showed significantly different genetic correlations between CanUD and cannabis use, whereas drinks per week (DPW) did not show differences in correlation [17]. Recent work has also demonstrated a high genetic correlation between opioid use disorder (OUD) and CanUD (r_g_= 0.78 ± 0.06, *p* = 3.8 × 10^−36^) and a moderate genetic correlation of OUD with cannabis use (r_g_= 0.22 ± 0.08, *p* = 3.8 × 10^−3^) [9]. A moderate genetic correlation has been reported between CanUD and problematic alcohol use (PAU) (r_g_= 0.61 ± 0.04, *p* = 4.9 × 10^−63^) [18].

**Fig. 1:**
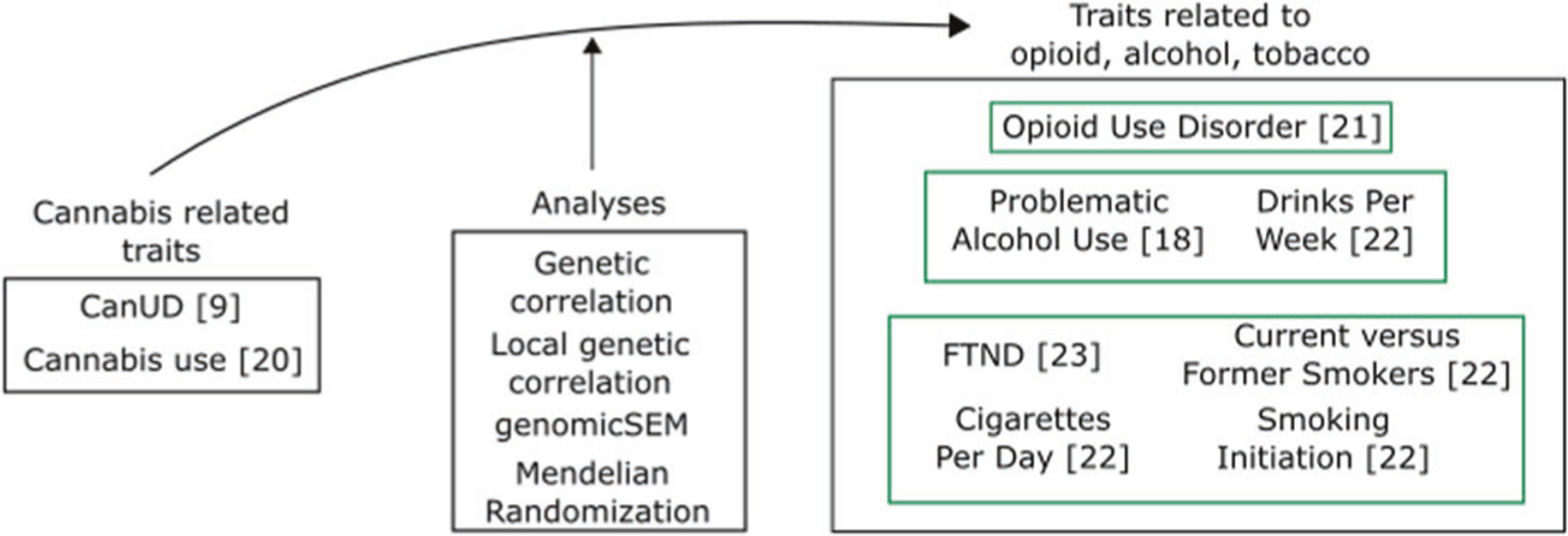
Schematic representation of traits and analyses.

Cannabis has sometimes been termed a “gateway” drug, thought to lead to other, potentially even more serious, substance dependencies. The phenotypic association observed between CanUD and other SUDs could also be attributable to common genetic liability. It is important to test this hypothesis using multiple methods, and to understand the biological relationships that underlie these relationships. A recent genome-wide association study (GWAS) of CanUD increased the number of known genomewide significant risk loci from 2 to 25 [9]. Making use of these data allows for greatly increased power in downstream genomics analyses concerning CanUD and new opportunities to understand the biology of CanUD and its relationship to other substance use traits. Accordingly, we investigated the relationship between CanUD and cannabis use with other SUDs and substance use traits related to opioids, alcohol, and tobacco (Fig. 1).

Using publicly available GWAS summary statistics, we first examined “global” genetic correlations between CanUD and cannabis use with other SUDs and substance use traits. We then performed local genetic correlation analyses to infer which part of the genome could be involved for both CanUD and cannabis use with the other SUDs and substance use traits. We also performed genomic structural equation modeling (genomic-SEM) among the included substance-related traits to understand their relationships and connections better. Mendelian randomization (MR) analyses were then conducted to investigate the causality of the relationship between genetic risk for CanUD and cannabis use with genetic risk for the other SUDs and substance use traits.

## METHODS

### Genetic correlation and traits

We performed genetic correlation analyses using linkage disequilibrium score regression [19], which uses GWAS summary statistics of the analyzed phenotypes as input. Based on European ancestry (EUR) subjects, we calculated the genetic correlations between CanUD [9] and cannabis use [20] with other SUDs and substance use traits related to opioids, alcohol, and tobacco (Supplementary Table 1). Specifically, for opioids, we used OUD [21]. For alcohol, we investigated PAU [18] (which is a proxy for alcohol use disorder) and DPW [22] (a quantity/frequency of use measure). For nicotine and tobacco, we used FTND scores [23], cigarettes per day [22], current versus former smokers [22], and smoking initiation [22]. Results between CanUD and cannabis use with FTND were reported previously [9] and are quoted herein for context. Power considerations restricted all of these analyses to European-ancestry populations.

We calculated significantly different correlations between CanUD and cannabis use using the formula of $p=\text{2*pnorm}\left(-\left|\text{Z}\right|\right)$, where pnorm provides the value of the cumulative density function of the normal distribution of the $Z=\frac{{\text{rg}}_{\text{CanUD}}-{\text{rg}}_{\text{CanUse}}}{\sqrt{{\text{se}}_{\text{CanUD}}^{2}+{\text{se}}_{\text{CanUse}}^{2}}}$, with a mean = 0, and the standard deviation = 1. We used a Bonferroni *p* value threshold = 0.0071 (0.05/7).

### Local genetic correlation

We performed LAVA [24] to infer genomic regions with a significant local genetic correlation for CanUD and cannabis use with the SUDs and substance use traits related to opioid, alcohol, and tobacco. Regions were defined by splitting the whole genome into 2495 blocks of around 1 MB to minimize the linkage disequilibrium (LD) between the regions [24]. For each region we initially calculated local genetic heritability for each trait with a Bonferroni-corrected significance threshold of 0.05/2,495 (*p* = 2.0 × 10^−5^). Then, we used the loci showing significant local genetic heritability to perform local genetic correlation tests. For CanUD this procedure resulted in 795 tests. For cannabis use, we performed 2938 tests. This resulted in two different Bonferroni-corrected *p* value thresholds, 0.05/795 (*p* = 6.3 × 10^−5^) for CanUD and 0.05/2,938 (*p* = 1.7 × 10^−5^) for cannabis use, to determine statistical significance.

We also carried out an independent correlation analysis between CanUD and cannabis use, which resulted into 487 tests, with a Bonferroni-corrected *p* value thresholds, 0.05/487 (*p* = 1.0 × 10^−4^).

### Genomic structural equation modeling

Genomic structural equation modeling (genomicSEM) [25] was used to examine the genomic architecture across CanUD, cannabis use, and the included set of related SUDs and substance use traits. Exploratory Factor Analysis (EFA) was used to assess model fit for models consisting of 1–5 factors. Confirmatory Factor Analysis (CFA) was then used to evaluate factor loadings and model fit as indicated by conventional model fit indices. Traits with EFA factor loadings >0.30 were allowed to load or co-load on the respective factors specified in the CFA. 1000 Genomes Phase 3 [26] European ancestry data were used as a reference panel. All models were specified using standard genomicSEM parameters [25].

### Mendelian randomization

MRlap [27] was used to conduct MR analyses to infer causality between CanUD and cannabis use with the traits related to opioids, alcohol, and tobacco. The choice of using MRlap was driven by its feature of being able to use potentially overlapping samples, which is a characteristic of many of the datasets included in the present analysis. As output, MRlap produces both observed and biases-corrected results. These latter results should be preferred when they are significantly different from the observed effects. We ran the inverse variance weighted (IVW) method implemented in MRlap, with a *p* value thresholds = 10^−5^ to select MR instruments, and LD threshold of 0.05 used for pruning MR instruments.

## RESULTS

### Genetic correlations among traits

**Fig. 2:**
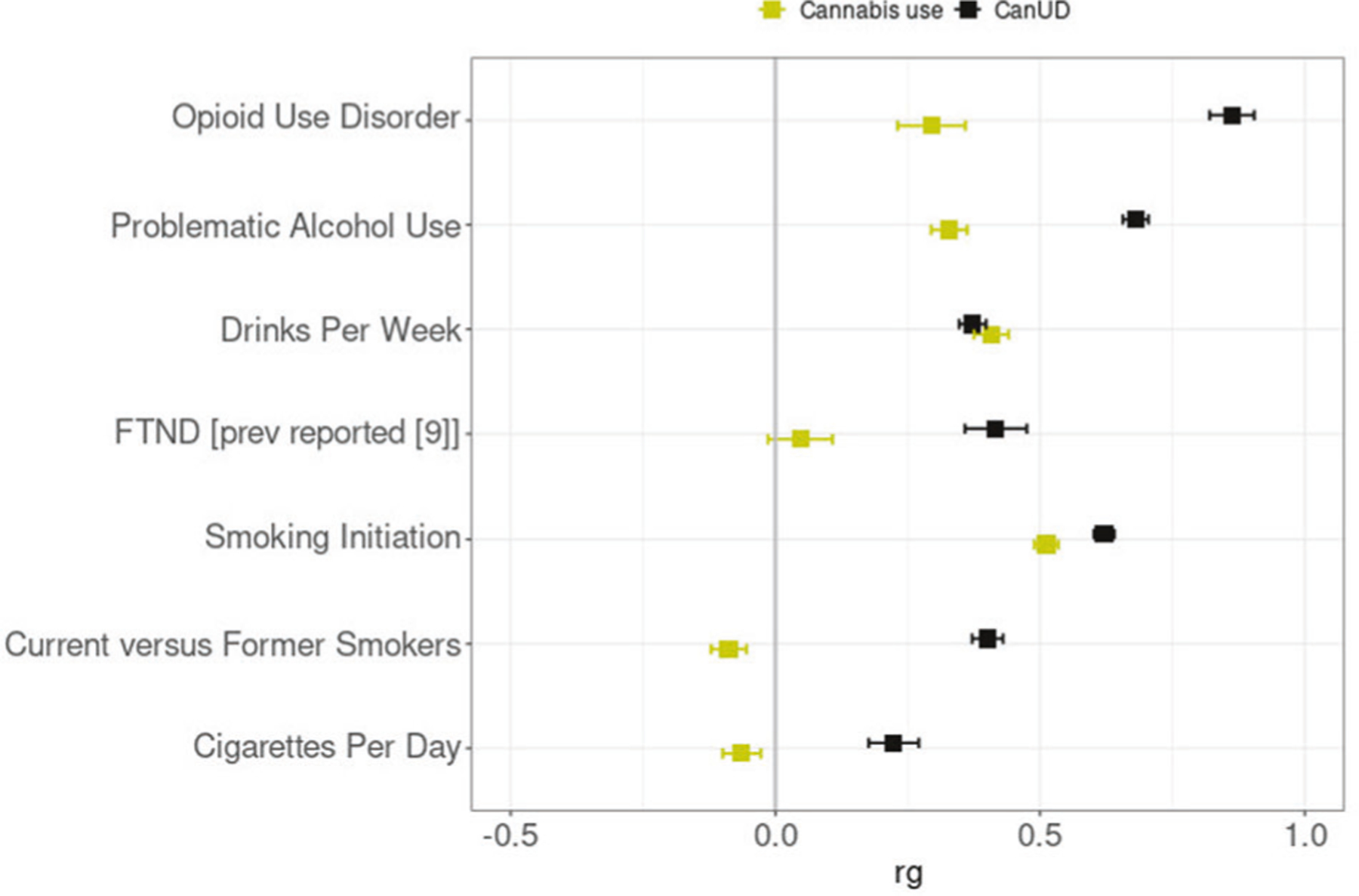
Genetic correlations. Genetic correlation (r_g_) analyses of CanUD and cannabis use with disorders and behaviors related to opioids, alcohol, and nicotine/tobacco. All the traits except DPW showed a significantly different correlation between CanUD and cannabis use.

From the genetic correlation analyses, we observed significant positive genetic correlations between CanUD with each of the analyzed traits. The highest genetic correlation was between CanUD and OUD (r_g_= 0.863 ± 0.042; *p* = 2.5 × 10^−93^), followed by PAU (r_g_= 0.681 ± 0.024; *p* = 7.3 × 10^−176^), and smoking initiation (r_g_= 0.621 ± 0.020; *p* = 1.7 × 10^−220^) (Fig. 2, Supplementary Table 2). For cannabis use, there was a different pattern, with substantial differences involving the traits related to smoking and tobacco. We found a significantly different genetic correlation between CanUD and cannabis use for all the traits except DPW (Fig. 2, Supplementary Table 1). We did not find significant genetic correlations between cannabis use with FTND, consistent with previous reports [9]. In our work, null results were also found between cannabis use and current versus former smokers, and cigarettes per day, respectively. Compared to CanUD, cannabis use also showed a genetic correlation much lower in magnitude with OUD (r_g_= 0.295 ± 0.064; *p* = 4.1 × 10^−6^) and PAU (r_g_= 0.328 ± 0.034; *p* = 4.1 × 10^−22^).

### Local genetic correlation

Local genetic correlation analyses found 11 regions with significant positive local genetic correlations between CanUD and smoking initiation (Supplementary Table 3). Nine significant local genetic correlations were found between CanUD and PAU. Even in the presence of a positive “global” genetic correlation between CanUD and PAU, 7 regions showed a negative local genetic correlation, whereas only 2 showed a positive local genetic correlation. We found 3 regions with a positive local genetic correlation between CanUD and DPW. FTND and OUD each showed one region with a positive local genetic correlation with CanUD, whereas cigarettes per day only had a single significant region showing a negative local genetic correlation with CanUD. Cannabis use also had several regions associated with smoking initiation, 13 with a significant positive local genetic correlation and 1 with a significant negative local genetic correlation (Supplementary Table 4). Two of these regions, chr3:84698481–85807679 and chr11:112755447–113889019 which includes *DRD2* (dopamine D2 receptor) (Supplementary Fig. 1), also had a significant positive local genetic correlation between smoking initiation and CanUD. Locus chr3:84698481–85807679, which includes the genes *CADM2* (cell adhesion molecule 2), *SNORA95* (small nucleolar RNA, H/ACA box 95), *MIR5688* (microRNA 5688), and *CADM-AS2* (CADM2 antisense RNA 2), showed also significant positive local genetic correlation for both cannabis use and CanUD with DPW.

The local genetic correlation analysis between CanUD and cannabis use provided six significant regions, four and two with significant positive and negative local genetic correlation, respectively (Supplementary Table 5).

### Genomic structural equation modeling

Of the EFA models examined, a four-factor model fit the data best. The four-factor model explained 0.83 of the cumulative variance. The respective factors each accounted for a meaningful proportion of overall variance (variance explained: 0.13–0.31) and all had sum of squared (SS) loadings greater than 1 (Supplementary Table 6).

**Fig. 3:**
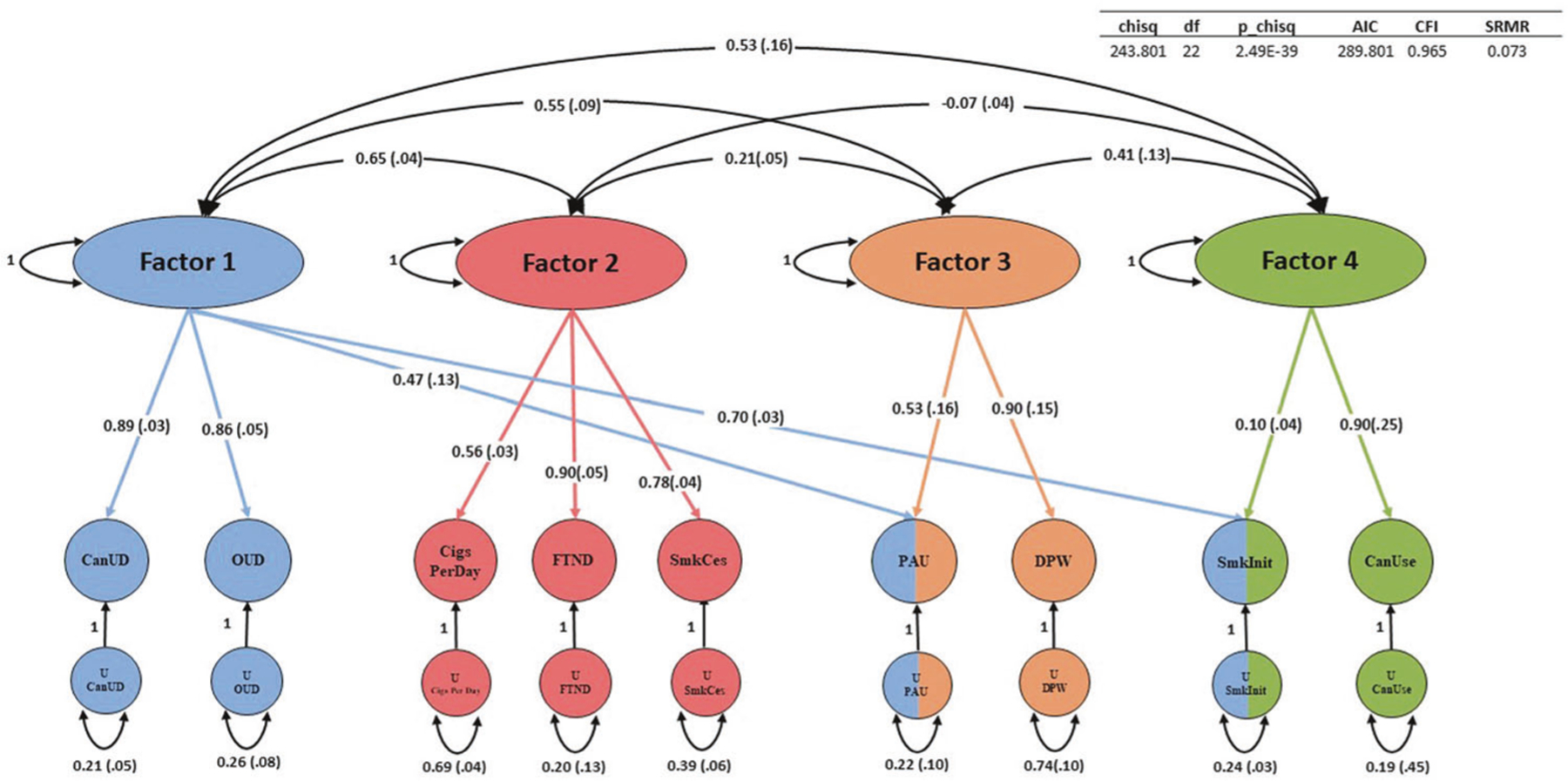
genomicSEM. GenomicSEM was used to cluster CanUD, cannabis use (CanUse), and the other seven traits related to opioids, alcohol, and nicotine/tobacco. Abbreviations (left to right) - OUD Opioid use disorder, CigsPerDay cigarettes per day, FTND Fagerström Test for Nicotine Dependence, SmkCes current versus former smokers, PAU Problematic Alcohol Use, DPW Drinks per week, SmkInit smoking initiation, Can Use, cannabis use.

The four-factor CFA fit the data well (comparative fit index = 0.97; *χ*^2^ = 243.80; Aikake information criterion = 289.80; standardized root mean square residual = 0.07) (Fig. 3, Supplementary Table 6). CanUD loaded most strongly on Factor 1 (loading = 0.89 ± 0.03) along with OUD (loading = 0.86 ± 0.05), PAU (loading = 0.47 ± 0.13), and smoking initiation (loading = 0.70 ± 0.03). PAU and smoking initiation each also co-loaded on other factors. PAU co-loaded on Factor 3 (loading = 0.53 ± 0.16) along with DPW (loading = 0.90 ± 0.15); smoking initiation co-loaded on Factor 4 (loading = 0.10 ± 0.04) along with cannabis use (loading = 0.90 ± 0.25). FTND (loading = 0.90 ± 0.05), Cigarettes per day (loading = 0.56 ± 0.03), and smoking cessation (loading = 0.78 ± 0.04) loaded together most strongly on Factor 2.

### Mendelian randomization

We initially found a bidirectional relationship between CanUD and cannabis use (effect_corrected_= 0.222 ± 0.035; *p* value_corrected_ = 2.9 × 10^−10^ considering CanUD as exposure, effect_corrected_= 0.173 ± 0.047; *p* value_corrected_ = 2.3 × 10^−4^ considering cannabis use as exposure).

**Fig. 4:**
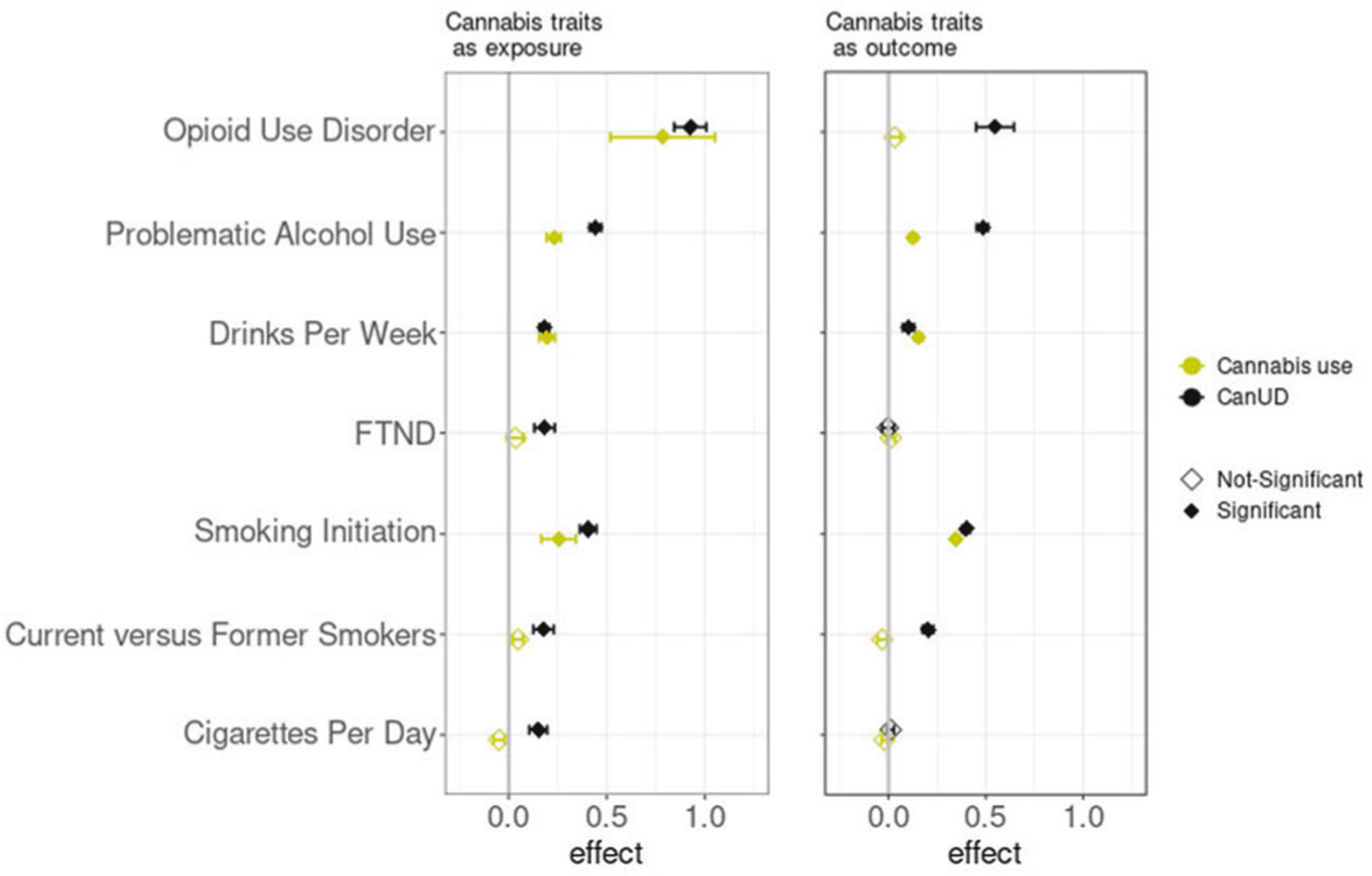
Mendelian randomization analyses. Mendelian Randomization (MR) analyses (*p* value threshold = 10^−5^) of CanUD and cannabis use as exposure versus disorders and behaviors related to opioids, alcohol, and nicotine/tobacco. CanUD and cannabis use are represented as exposures on the left panel, and as outcome on the right panel.

MR analyses inferred significant causal effects of CanUD on all of the analyzed traits, when using a *p* value threshold of 10^−5^ for defining genetic instruments (Fig. 4, Supplementary Tables 7–8). Moreover, MR analyses showed a bidirectional relationship between CanUD with OUD, PAU, smoking initiation, current versus former smokers, and DPW. The strongest causal effect of CanUD on other disorders was shown for OUD (effect_corrected_= 0.925 ± 0.082; *p* value_corrected_ = 9.2 × 10^−30^) followed by PAU (effect_corrected_= 0.443 ± 0.030; *p* value_corrected_ = 5.9 × 10^−50^). For cannabis use, MR analyses detected bidirectional relationships with PAU, smoking initiation, and DPW (Fig. 4, Supplementary Table 9–10). Cannabis use was also associated with higher risk of developing OUD (effect_corrected_= 0.785 ± 0.266; *p* value_corrected_ = 3.6 × 10^−3^), although the effect was weaker compared to CanUD.

## DISCUSSION

In the present study we performed a series of genetic analyses to understand better the differing relationships between CanUD and cannabis use with other SUDs and substance use traits. This work has only recently become possible because of substantial advancements in GWAS power for CanUD, with a recent GWAS that identified numerous significant associations [9]. This GWAS provided us with the genetic instruments necessary to study causal relationships with other traits. We found a significant positive genetic correlation between CanUD and current versus former smokers. OUD, PAU, and smoking initiation showed significant positive genetic correlations with cannabis use. We previously reported positive genetic correlations between CanUD and other substance-related traits [9, 17, 18], and the present results are consistent with these prior analyses. It is often seen that substance use disorder traits are different genetically from substance use/quantity-frequency traits [28] and this is true specifically for cannabis traits [9, 29, 30]. Consistent with this expected pattern, we found significantly different genetic correlations with the range of traits studied here between CanUD and cannabis use. The only trait that did not show a significant genetic difference between CanUD and cannabis use was DPW, also consistent with previous findings [17].

We used genomicSEM to focus on the genetic architecture of a range of substance use and SUD traits in greater detail. Our analysis resulted in a four-factor solution—one factor including SUDs; one factor specific to alcohol-related traits; a factor including heavy tobacco smoking traits (FTND, cigarettes per day, and current versus former smokers); and a factor indexing lifetime ever-use (smoking initiation and lifetime cannabis use). A few notable findings arise here including that smoking initiation appears to be related to both the SUD factor and the lifetime ever-use factor—expected, as regular use of a substance must be initiated in order for a SUD to develop. Also FTND is not included on the SUD factor and is loaded more strongly with other traits indexing heavy tobacco use (such as cigarettes per day [a question also contained within the FTND assessment] and current versus former smokers [i.e., smokers that used tobacco to a point that they eventually quit]). PAU co-loaded with other SUDs and the alcohol-specific factor. The current analysis provides superior resolution in terms of substance use traits and builds on previous work that focused on contextualizing CanUD amongst a broader array of trait domains including factors capturing functional impairment, impulsivity and risk taking, psychopathology, and substance dependence [9].

MR analyses were then used to investigate the causality between CanUD and cannabis use with the other discussed traits. We found evidence of bidirectional causality between CanUD and OUD, whereas for cannabis use, we found that the use of cannabis only increases the risk of OUD and not vice versa.

As previously suggested in studies of phenotype, users of cannabis were more likely to develop OUD than non-users of cannabis: a meta-analysis including 102,461 individuals from the United States, Australia, and New Zealand reported an odds ratio (OR) = 2.76 of transitioning from cannabis to opioid use compared to non-cannabis users, and an OR = 2.52 of transitioning from opioid use to OUD given prior cannabis use [31]. Also, the use of cannabis increased the risk of developing incident nonmedical prescription OUD (OR = 7.76) [32]. We found genetic evidence that cannabis use and CanUD increase the risk of OUD, suggesting that future risk of OUD may be an important harmful possible outcome of cannabis intake.

Regarding traits related to alcohol use, we found bidirectional causality between CanUD and PAU. PAU is a phenotype which includes alcohol dependence, AUDIT-P (Alcohol Use Disorders Identification Test-Problem Score [33]), and alcohol use disorder (AUD) [18]. A similar phenotypic relationship was seen in a previous study of 127 veterans who reported at least one day of marijuana and alcohol co-use in the past 180 days, which showed evidence of heavy drinking (≥5 drinks for men and ≥4 drinks per women in a day) during the use of marijuana for individuals with co-occurring AUD and CanUD (OR = 2.51) or AUD only (OR = 1.91), in comparison to moderate drinking (at least one drink in a day) [34]. We also found bidirectional causality between CanUD and DPW. For cannabis use, as for CanUD, we found bidirectional causality with PAU and DPW. These results are consistent with a phenotypic analysis showing that the use of cannabis increased the probability to develop alcohol use disorder (OR = 5.43) compared to cannabis abstainers [35].

For traits related to tobacco use, MR analyses revealed bidirectional causality between CanUD and smoking initiation and current versus former smokers, whereas there was a unidirectional causality of CanUD increasing cigarettes per day and FTND. For cannabis use, we found bidirectional causality with smoking initiation.

Tobacco smokers have high risk of developing CanUD, with smokers being 8.3 times more likely to develop CanUD compared to non-smokers [36]. Quit ratios for individuals with CanUD have been reported as being less than half the quit ratios of those without CanUD [37], and individuals with earlier initiation of tobacco use are more likely to report a lifetime CanUD [38].

The local genetic correlation results illustrate another aspect of the genetic factors shared between smoking initiation with cannabis use (14 significant local genetic correlations) and CanUD (11 significant local genetic correlations). Of the two regions showing significant local genetic correlation for both cannabis use and CanUD with smoking initiation, chr3:84698481–85807679 includes the gene C*ADM2* (cell adhesion molecule 2), which is known to play a role in cannabis use, lifetime smoking, regular alcohol use, and also impulsivity, risky behavior, and physical activity [39–41]. The second region, chr11:112755447–113889019, includes several genes among which is *DRD2*, which has been shown to be associated to numerous SUD traits and to schizophrenia, among other psychiatric traits [29].

In summary, we report here data regarding the relationship between CanUD and cannabis use with a set of substance use and use disorder traits, noting characteristic differences between substance use and SUDs. They were initially highlighted as significantly different genetic correlations. This observation is further supported by cannabis use and CanUD clustered on different factors in our genomicSEM, and underscored by MR analyses showing differing causal relationships, particularly for the cigarette smoking traits (FTND, current versus former smokers, and cigarettes per day) where risk was increased by CanUD but not by cannabis use.

This work has some limitations. First, it was possible to include only EUR subjects due to lack of sufficient GWAS summary statistics from other populations. Repeating the analyses with non-EUR ancestries would be important to understand the studied relationships of CanUD and cannabis use more fully. Second, we could not use multivariable Mendelian randomization (MVMR), which is a method to infer the causal effect of multiple exposures on an outcome [42], because of possible sample overlap. Instead, we performed two-sample MR analyses with MRlap to take sample overlap into account. MVMR analyses, had they been possible, would have allowed us to study the causal effect of CanUD and cannabis use together on the different outcomes. Third, secular trends such as changes in patterns of use attributable to legalization of cannabis in some locations and the development of higher potency cannabis products cannot be captured in our analyses.

In conclusion, we used a series of genetic approaches to evaluate the relationships and causality of CanUD and cannabis use on traits related to opioids, alcohol, and smoking. This work strongly supported phenotypic evidence of CanUD increasing the risk of developing other SUDs, and was often bidirectional. As we learn more about the biology of CanUD and subject it to greater scrutiny, we continue to discover novel harms that may result from cannabis use. For example, the causal relationship of cannabis use on OUD provides support for a role of cannabis as “gateway” drug. Here, we report that cannabis use traits increase risk for multiple other SUD traits, and that multiple SUD traits increase risk for cannabis use traits. With the increasing ambient availability of cannabis in our society, these mutually increased risks and harms can be expected to increase in importance and impact.

## Supplementary Material

Supplementary Material

Supplementary Tables

## References

[1] Hasin DS, Kerridge BT, Saha TD, Huang B, Pickering R, Smith SM, Prevalence and correlates of DSM-5 Cannabis Use Disorder, 2012–2013: findings from the national epidemiologic survey on alcohol and related conditions-III. Am J Psychiatry. 2016;173:588–99. 26940807 10.1176/appi.ajp.2015.15070907 PMC5026387

[2] McLellan AT. Substance misuse and substance use disorders: why do they matter in healthcare? Trans Am Clin Climatol Assoc 2017;128:112–30. 28790493 PMC5525418

[3] Gan WQ, Buxton JA, Scheuermeyer FX, Palis H, Zhao B, Desai R, Risk of cardiovascular diseases in relation to substance use disorders. Drug Alcohol Depend. 2021;229:109132. 34768052 10.1016/j.drugalcdep.2021.109132

[4] Chen M, Lu Y-l, Chen X-f, Wang Z, Ma L. Association of cannabis use disorder with cardiovascular diseases: a two-sample Mendelian randomization study. Front Cardiovasc Med 2022;9:966707. 36277767 10.3389/fcvm.2022.966707 PMC9582269

[5] Yusufov M, Braun IM, Pirl WF. A systematic review of substance use and substance use disorders in patients with cancer. Gen Hospital Psychiatry. 2019;60:128–36. 10.1016/j.genhosppsych.2019.04.016 31104826

[6] Sheikh Andalibi MS, Rezaei Ardani A, Amiri A, Morovatdar N, Talebi A, Azarpazhooh MR, The association between substance use disorders and long-term outcome of stroke: results from a population-based study of stroke among 450,229 urban citizens. Neuroepidemiology. 2021;55:171–9. 33975326 10.1159/000514401

[7] Thylstrup B, Seid AK, Tjagvad C, Hesse M. Incidence and predictors of drug overdoses among a cohort of >10,000 patients treated for substance use disorder. Drug Alcohol Depend. 2020;206:107714. 31753733 10.1016/j.drugalcdep.2019.107714

[8] Jones CM, McCance-Katz EF. Co-occurring substance use and mental disorders among adults with opioid use disorder. Drug Alcohol Depend. 2019;197:78–82. 30784952 10.1016/j.drugalcdep.2018.12.030

[9] Levey DF, Galimberti M, Deak JD, Wendt FR, Bhattacharya A, Koller D, Multiancestry genome-wide association study of cannabis use disorder yields insight into disease biology and public health implications. Nat Genet 2023;55:2094–103. 37985822 10.1038/s41588-023-01563-z PMC10703690

[10] Selamoglu A, Langley C, Crean R, Savulich G, Cormack F, Sahakian BJ, Neuropsychological performance in young adults with cannabis use disorder. J Psychopharmacol 2021;35:1349–55. 34694178 10.1177/02698811211050548 PMC8600580

[11] Compton WM, Saha TD, Conway KP, Grant BF. The role of cannabis use within a dimensional approach to cannabis use disorders. Drug Alcohol Depend. 2009;100:221–7. 19062204 10.1016/j.drugalcdep.2008.10.009 PMC3153503

[12] Connor JP, Stjepanović D, Le Foll B, Hoch E, Budney AJ, Hall WD. Cannabis use and cannabis use disorder. Nat Rev Dis Prim 2021;7:16. 33627670 10.1038/s41572-021-00247-4 PMC8655458

[13] Leung J, Chan GCK, Hides L, Hall WD. What is the prevalence and risk of cannabis use disorders among people who use cannabis? a systematic review and meta-analysis. Addic Behav 2020;109:106479. 10.1016/j.addbeh.2020.106479 32485547

[14] Roberts E What impact could the legalisation of recreational cannabis have on the health of the UK? Lessons from the rest of the world. Br J Psychiatry. 2024;224:117–8. 38268114 10.1192/bjp.2024.4 PMC7615739

[15] Zellers SM, Ross JM, Saunders GRB, Ellingson JM, Anderson JE, Corley RP, Impacts of recreational cannabis legalization on cannabis use: a longitudinal discordant twin study. Addiction. 2023;118:110–8. 36002928 10.1111/add.16016 PMC10086942

[16] Zellers S, Alexander J, Ellingson JM, Schaefer JD, Corley RP, Iacono W, Limited psychological and social effects of lifetime cannabis use frequency: Evidence from a 30-year community study of 4,078 twins. J Psychopathol Clin Sci 2024;133:115–28. 38147055 10.1037/abn0000867 PMC10751959

[17] Johnson EC, Demontis D, Thorgeirsson TE, Walters RK, Polimanti R, Hatoum AS, A large-scale genome-wide association study meta-analysis of cannabis use disorder. Lancet Psychiatry. 2020;7:1032–45. 33096046 10.1016/S2215-0366(20)30339-4 PMC7674631

[18] Zhou H, Kember RL, Deak JD, Xu H, Toikumo S, Yuan K, Multi-ancestry study of the genetics of problematic alcohol use in over 1 million individuals. Nat Med 2023;29:3184–92. 38062264 10.1038/s41591-023-02653-5 PMC10719093

[19] Bulik-Sullivan B, Finucane HK, Anttila V, Gusev A, Day FR, Loh P-R, An atlas of genetic correlations across human diseases and traits. Nat Genet 2015;47:1236–41. 26414676 10.1038/ng.3406 PMC4797329

[20] Pasman JA, Verweij KJH, Gerring Z, Stringer S, Sanchez-Roige S, Treur JL, GWAS of lifetime cannabis use reveals new risk loci, genetic overlap with psychiatric traits, and a causal effect of schizophrenia liability. Nat Neurosci 2018;21:1161–70. 30150663 10.1038/s41593-018-0206-1 PMC6386176

[21] Deak JD, Zhou H, Galimberti M, Levey DF, Wendt FR, Sanchez-Roige S, Genome-wide association study in individuals of European and African ancestry and multi-trait analysis of opioid use disorder identifies 19 independent genome-wide significant risk loci. Mol Psychiatry. 2022;27:3970–9. 35879402 10.1038/s41380-022-01709-1 PMC9718667

[22] Saunders GRB, Wang X, Chen F, Jang S-K, Liu M, Wang C, Genetic diversity fuels gene discovery for tobacco and alcohol use. Nature. 2022;612:720–4. 36477530 10.1038/s41586-022-05477-4 PMC9771818

[23] Quach BC, Bray MJ, Gaddis NC, Liu M, Palviainen T, Minica CC, Expanding the genetic architecture of nicotine dependence and its shared genetics with multiple traits. Nat Commun 2020;11:5562. 33144568 10.1038/s41467-020-19265-z PMC7642344

[24] Werme J, van der Sluis S, Posthuma D, de Leeuw CA. An integrated framework for local genetic correlation analysis. Nat Genet 2022;54:274–82. 35288712 10.1038/s41588-022-01017-y

[25] Grotzinger AD, Rhemtulla M, de Vlaming R, Ritchie SJ, Mallard TT, Hill WD, Genomic structural equation modelling provides insights into the multivariate genetic architecture of complex traits. Nat Hum Behav 2019;3:513–25. 30962613 10.1038/s41562-019-0566-x PMC6520146

[26] Auton A, Abecasis GR, Altshuler DM, Durbin RM, Abecasis GR, Bentley DR, A global reference for human genetic variation. Nature. 2015;526:68–74. 26432245 10.1038/nature15393 PMC4750478

[27] Mounier N, Kutalik Z. Bias correction for inverse variance weighting Mendelian randomization. Genet Epidemiol 2023;47:314–31. 37036286 10.1002/gepi.22522

[28] Gelernter J, Polimanti R. Genetics of substance use disorders in the era of big data. Nat Rev Genet 2021;22:712–29. 34211176 10.1038/s41576-021-00377-1 PMC9210391

[29] Jang S-K, Jiang Y, Liu M, Liu DJ, Saunders G, Vrieze S. Genetic correlation, pleiotropy, and causal associations between substance use and psychiatric disorder. Psychol Med 2020;52:968–78. 32762793 10.1017/S003329172000272X PMC8759148

[30] Abdellaoui A, Verweij KJH. Dissecting polygenic signals from genome-wide association studies on human behaviour. Nat Hum Behav 2021;5:686–94. 33986517 10.1038/s41562-021-01110-y

[31] Wilson J, Mills K, Freeman TP, Sunderland M, Visontay R, Marel C. Weeding out the truth: a systematic review and meta-analysis on the transition from cannabis use to opioid use and opioid use disorders, abuse or dependence. Addiction. 2022;117:284–98. 34264545 10.1111/add.15581

[32] Olfson M, Wall MM, Liu SM, Blanco C. Cannabis use and risk of prescription opioid use disorder in the United States. Am J Psychiatry. 2018;175:47–53. 28946762 10.1176/appi.ajp.2017.17040413 PMC5756122

[33] Sanchez-Roige S, Palmer AA, Fontanillas P, Elson SL, Adams MJ, Howard DM, Genome-wide association study meta-analysis of the Alcohol Use Disorders Identification Test (AUDIT) in two population-based cohorts. Am J Psychiatry. 2019;176:107–18. 30336701 10.1176/appi.ajp.2018.18040369 PMC6365681

[34] Metrik J, Gunn RL, Jackson KM, Sokolovsky AW, Borsari B. Daily patterns of marijuana and alcohol co-use among individuals with alcohol and cannabis use disorders. Alcohol: Clin Exp Res 2018;42:1096–104. 29656401 10.1111/acer.13639 PMC5984172

[35] Weinberger AH, Platt J, Goodwin RD. Is cannabis use associated with an increased risk of onset and persistence of alcohol use disorders? A three-year prospective study among adults in the United States. Drug Alcohol Depend. 2016;161:363–7. 26875671 10.1016/j.drugalcdep.2016.01.014 PMC5028105

[36] Agrawal A, Budney AJ, Lynskey MT. The co-occurring use and misuse of cannabis and tobacco: a review. Addiction. 2012;107:1221–33. 22300456 10.1111/j.1360-0443.2012.03837.x PMC3397803

[37] Weinberger AH, Pacek LR, Wall MM, Gbedemah M, Lee J, Goodwin RD. Cigarette smoking quit ratios among adults in the USA with cannabis use and cannabis use disorders, 2002–16. Tob Control. 2020;29:74–80. 30952691 10.1136/tobaccocontrol-2018-054590 PMC12628350

[38] Millar SR, Mongan D, Smyth BP, Perry IJ, Galvin B. Relationships between age at first substance use and persistence of cannabis use and cannabis use disorder. BMC Public Health. 2021;21:997. 34044802 10.1186/s12889-021-11023-0 PMC8157747

[39] Arends RM, Pasman JA, Verweij KJH, Derks EM, Gordon SD, Hickie I, Associations between the CADM2 gene, substance use, risky sexual behavior, and self-control: a phenome-wide association study. Addict Biol 2021;26:e13015. 33604983 10.1111/adb.13015 PMC8596397

[40] Sanchez-Roige S, Jennings MV, Thorpe HHA, Mallari JE, van der Werf LC, Bianchi SB, CADM2 is implicated in impulsive personality and numerous other traits by genome- and phenome-wide association studies in humans and mice. Transl Psychiatry. 2023;13:167. 37173343 10.1038/s41398-023-02453-y PMC10182097

[41] Klimentidis YC, Raichlen DA, Bea J, Garcia DO, Wineinger NE, Mandarino LJ, Genome-wide association study of habitual physical activity in over 377,000 UK Biobank participants identifies multiple variants including CADM2 and APOE. Int J Obes 2018;42:1161–76. 10.1038/s41366-018-0120-3 PMC6195860 29899525

[42] Rasooly D, Peloso GM. Two-sample multivariable mendelian randomization analysis using R. Curr Protoc 2021;1:e335. 34936225 10.1002/cpz1.335 PMC8767787

