# Supplementary material for: Genetic influences and causal pathways shared between cannabis use disorder and other substance use traits
Source: Mol Psychiatry. 2024 Apr 5;29(9):2905–10. doi: 10.1038/s41380-024-02548-y (PMC11419938; doi:10.1038/s41380-024-02548-y)
Supplement: Supplementary file 1 — Supplementary Material [file 41380_2024_2548_MOESM1_ESM.docx]

**Supplementary Information - Genetic influences and causal pathways shared between cannabis use disorder and other substance use traits**

Marco Galimberti^1,2^, Daniel F. Levey^1,2^, Joseph D. Deak^1,2^, Hang Zhou^1,2^, Murray B. Stein^3,4^, Joel Gelernter^1,2,5*^

1: Department of Psychiatry, Yale University School of Medicine, New Haven, CT, USA

2: Veterans Affairs Connecticut Healthcare System, West Haven, CT, USA

3: Department of Psychiatry and School of Public Health, University of California San Diego, La Jolla, CA, USA

4: VA San Diego Healthcare System, San Diego, CA, USA

5: Departments of Genetics and Neuroscience, Yale University School of Medicine, New Haven, CT, USA

*: corresponding author

**Supplementary Figures**


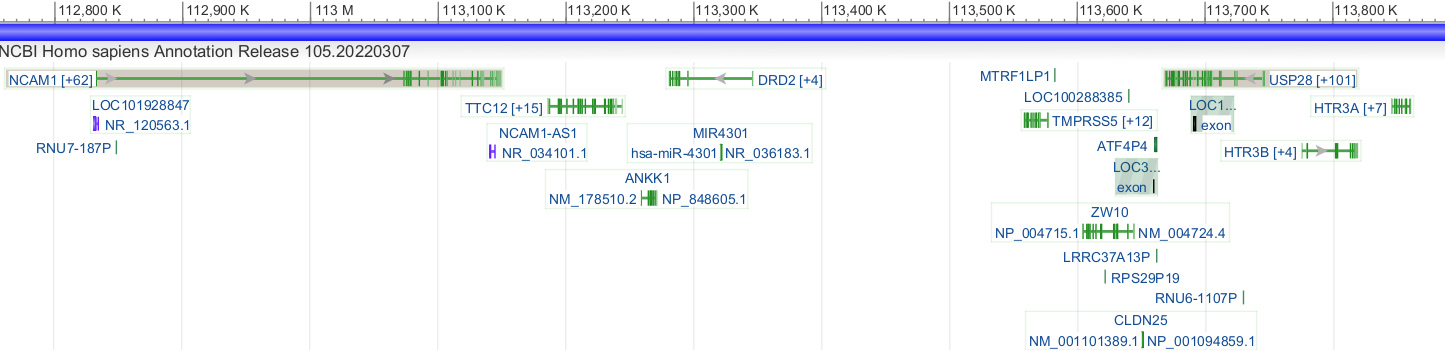


**Supplementary Fig 1. | View of locus chr11:112755447-113889019**. NIH Genome Data Viewer of locus chr11:112755447-113889019, using genome assembly GRCh37.p13.
